# A Comparison of the Epidemiology and Clinical Presentation of Seasonal Influenza A and 2009 Pandemic Influenza A (H1N1) in Guatemala

**DOI:** 10.1371/journal.pone.0015826

**Published:** 2010-12-30

**Authors:** Kim A. Lindblade, Wences Arvelo, Jennifer Gray, Alejandra Estevez, Gal Frenkel, Lissette Reyes, Fabiola Moscoso, Juan Carlos Moir, Alicia M. Fry, Sonja J. Olsen

**Affiliations:** 1 Division of Global Disease Detection and Emergency Response, Centers for Disease Control and Prevention, Atlanta, Georgia, United States of America; 2 Centers for Disease Control and Prevention Regional Office for Central America and Panama, Guatemala City, Guatemala; 3 Center for Health Studies, Universidad del Valle de Guatemala, Guatemala City, Guatemala; 4 Field Epidemiology Training Program, Ministry of Public Health and Social Assistance, Guatemala City, Guatemala; 5 Ministry of Public Health and Social Assistance, Guatemala City, Guatemala; 6 Influenza Division, Centers for Disease Control and Prevention, Atlanta, Georgia, United States of America; The University of Hong Kong, Hong Kong

## Abstract

**Background:**

A new influenza A (H1N1) virus was first found in April 2009 and proceeded to cause a global pandemic. We compare the epidemiology and clinical presentation of seasonal influenza A (H1N1 and H3N2) and 2009 pandemic influenza A (H1N1) (pH1N1) using a prospective surveillance system for acute respiratory disease in Guatemala.

**Methodology/Findings:**

Patients admitted to two public hospitals in Guatemala in 2008–2009 who met a pneumonia case definition, and ambulatory patients with influenza-like illness (ILI) at 10 ambulatory clinics were invited to participate. Data were collected through patient interview, chart abstraction and standardized physical and radiological exams. Nasopharyngeal swabs were taken from all enrolled patients for laboratory diagnosis of influenza A virus infection with real-time reverse transcription polymerase chain reaction. We identified 1,744 eligible, hospitalized pneumonia patients, enrolled 1,666 (96%) and tested samples from 1,601 (96%); 138 (9%) had influenza A virus infection. Surveillance for ILI found 899 eligible patients, enrolled 801 (89%) and tested samples from 793 (99%); influenza A virus infection was identified in 246 (31%). The age distribution of hospitalized pneumonia patients was similar between seasonal H1N1 and pH1N1 (*P = *0.21); the proportion of pneumonia patients <1 year old with seasonal H1N1 (39%) and pH1N1 (37%) were similar (*P = *0.42). The clinical presentation of pH1N1 and seasonal influenza A was similar for both hospitalized pneumonia and ILI patients. Although signs of severity (admission to an intensive care unit, mechanical ventilation and death) were higher among cases of pH1N1 than seasonal H1N1, none of the differences was statistically significant.

**Conclusions/Significance:**

Small sample sizes may limit the power of this study to find significant differences between seasonal influenza A and pH1N1. In Guatemala, influenza, whether seasonal or pH1N1, appears to cause severe disease mainly in infants; targeted vaccination of children should be considered.

## Introduction

In April 2009, a new influenza A (H1N1) virus with a unique combination of gene segments not previously identified among influenza viruses was reported from the United States and Mexico [1]. Immediately after the discovery of 2009 pandemic influenza A (H1N1) (pH1N1), there were many reports comparing pH1N1 with previous influenza seasons as a way to predict the clinical presentation of cases, the severity of the pandemic and high-risk groups [1]–[3]. Studies eventually identified a number of important differences between seasonal influenza and pH1N1, including a younger age distribution [4], novel risk factors such as obesity [5]–[7], and symptoms previously not frequently associated with influenza infection, such as diarrhea and vomiting [8]–[10]. Other reports found similarities between pH1N1 and seasonal influenza with regard to basic reproduction number [11], range of severity [8], clinical symptoms of hospitalized patients [2], and risk factors for severe disease [12].

Comparisons of the epidemiology and clinical presentation of seasonal influenza and pH1N1 can be complicated by the use of case series from different years, as this might introduce biases due to changes in practices or procedures as a result of the pandemic. There have been few reports of concurrent comparisons of the epidemiology of seasonal influenza and pH1N1 [13]–[18], and only one from a low-resource setting in the tropics where, until recently, influenza was not recognized as a significant problem [19]–[21]. The 2009 influenza pandemic has helped to raise the profile of influenza as an important cause of morbidity and mortality in tropical developing countries but there is insufficient data from resource-limited countries in the tropics.

In Guatemala, we have been conducting prospective, population-based surveillance for severe acute respiratory disease and influenza-like illness (ILI) since 2007. We recently reported on the epidemiologic and clinical presentation of pH1N1 and described a younger population than that affected by this virus in other parts of the world [22]. In this paper, we present a comparison of the clinical presentation of pH1N1, seasonal influenza A (H1N1) and seasonal influenza A (H3N2) in a resource-limited country in the tropics.

## Methods

### Human subjects

All patients 18 years of age or older were asked for verbal consent for screening and, if they met the case definition, written, informed consent to participate in the surveillance study. Relatives of adult patients who were unconscious or unable to provide consent on enrollment were asked to provide written, informed consent for their relative to participate, and this consent was renewed directly with the patient on regaining consciousness. Parents or guardians of children <18 years old were asked for verbal consent to screen their child to determine eligibility, after which written, informed consent was requested from the parents or guardians and written, informed assent from children aged 7 to 17 years old. The protocol received approval from the institutional review boards of the Universidad del Valle de Guatemala (UVG; Guatemala City, Guatemala) and Centers for Disease Control and Prevention (CDC; Atlanta, GA) and approval from the Guatemalan Ministry of Public Health and Social Welfare (MSPAS; Guatemala City, Guatemala). All positive pH1N1 results were reported immediately to the MSPAS, who informed the local public health authorities and patients in each site. The policy of the MSPAS was to provide oseltamivir treatment free of charge to all patients confirmed with pH1N1, although few patients received timely antiviral treatment due to limited stocks and delays in reporting results.

### Study area and design

Guatemala, with a population over 14 million, has a gross national income per capita of $2680 and is considered a middle-income country by the World Bank (http://data.worldbank.org/indicator/NY.GNP.PCAP.CD, accessed on 1 September 2010). Seasonal influenza vaccination is part of the national immunization schedule for persons ≥50 years old and healthcare workers, but coverage among these groups is low despite the vaccine being provided free of charge when available.

A description of the surveillance system for hospitalized pneumonia and ambulatory ILI in Guatemala has been presented previously [22]. Briefly, we conducted prospective surveillance for hospitalized pneumonia and ILI in the Departments of Santa Rosa (total population: 319,963) and Quetzaltenango (total population: 705,301). In Santa Rosa, surveillance for hospitalized pneumonia began in November 2007 and was conducted at the only hospital in the department, the National Hospital of Cuilapa. This hospital is a 176-bed regional referral hospital with a four-bed pediatric intensive care unit (ICU) and a four-bed adult ICU. In Quetzaltenango, surveillance for hospitalized pneumonia began in February 2009 and was conducted at the Western Regional Hospital, one of two general-purpose public hospitals in the department. The Western Regional Hospital is a larger facility with 425 beds, including 22 pediatric and six adult ICU beds. Both hospitals provide free care and serve mostly low- and mid-income populations.

Surveillance for ILI in public ambulatory clinics began in Santa Rosa in November 2007 in one health center (staffed by at least one physician) and was then expanded to five additional health posts (staffed by nurses) in June 2009 in response to the pH1N1 pandemic. Surveillance for ILI in ambulatory clinics in Quetzaltenango began in July 2009 in three health centers and one health post. Health care is also provided free of charge at these ambulatory clinics.

Prior to the pandemic, surveillance was limited to residents of the catchment area of each facility, but this geographic restriction was lifted in May 2009 to assist with monitoring the pandemic.

### Case definitions

A case of pneumonia was defined as a patient admitted to the hospital with at least one sign of acute infection and at least one respiratory sign or symptom from the respective columns in Table 1. A case of ILI was defined according to PAHO/CDC guidelines as a patient presenting to an ambulatory health clinic with a measured temperature >38°C and either cough or sore throat [23]. Suspect cases were identified prospectively by study nurses through review of ward registers or patient chief complaints to find any patients with respiratory-related illnesses. In addition, because this surveillance system also collects information on gastrointestinal, neurological and febrile illnesses, patients admitted for, or presenting with, complaints related to these syndromes were also screened to determine whether they met the case definition for pneumonia (hospital) or ILI (ambulatory clinic).

**Table 1:** Pneumonia Case Definition*, Guatemala, 2008-2009.

Signs of acute infection	Symptoms of respiratory disease
Fever (≥38°C)	Tachypnea
Hypothermia (<35.5°C)	<2 months: ≥60 respiration rate (RR)
Abnormal white blood cell count (WBC)	2 to 11 months: ≥50 RR
<5 years: <5500 or >15000	12 to 59 months: ≥40 RR
≥5 years: <3000 or >11000	5 years and older: ≥20 RR
Abnormal white blood cell differential	Cough
	Sputum production
	Pleuritic chest pain
	Hemoptysis
	Difficulty breathing
	Shortness of breath
	Sore throat
	For children <2 years old only
	Child pauses repeatedly while breastfeeding or drinking
	Chest indrawing
	Nasal flaring
	Noisy breathing

*Pneumonia case definition: at least one sign of acute infection and at least one symptom of respiratory disease.

### Data and sample collection

In the hospital, clinical and epidemiologic data were collected from both patient interviews and chart reviews. The highest temperature (axillary) documented in the first 24 hours after admission was recorded. A standard review of chest radiographs was undertaken by a study radiologist, and a standard pulmonary physical exam was performed by a study physician. Oxygen saturation was measured by study nurses with a pulse oximeter. Patients were asked whether they had been diagnosed with any of the following chronic conditions: asthma, other lung disease, diabetes, cancer, chronic cardiovascular disease (hypertension or heart disease), liver disease, kidney disease or any immunocompromised condition (including HIV/AIDS).

In the ambulatory clinics, axillary temperature was measured by study nurses and all other clinical and epidemiologic data were collected through patient interview. In both the hospital and ambulatory clinics, care sought for the current illness episode prior to hospital admission or presentation to the ambulatory clinic was reported by the patient or caregiver, along with any medicines taken prior to admission or presentation. Diarrhea was defined as three or more liquid or loose stools in a 24-hour period during the last seven days. Trained study nurses took nasopharyngeal swabs (NP) from all eligible and consenting patients with pneumonia and ILI, whereas oropharyngeal (OP) swabs were also taken from pneumonia patients; NP and OP swabs were put into one tube with viral transport media and stored at 4°C until they could be processed and sent to the laboratory at the UVG.

All patients with hospitalized pneumonia were asked to return three to six weeks after discharge for a follow-up visit. Patients who did not return by six weeks were followed with a phone call to determine their vital status.

### Laboratory diagnostics

A laboratory-confirmed case of influenza A was defined as a case of pneumonia or ILI with influenza A virus infection as determined by real-time reverse transcription polymerase-chain reaction (rRT-PCR). Subtyping for influenza A to differentiate viruses as seasonal H1N1 and H3N2 and pH1N1 was conducted using a standardized CDC protocol [24]. We tested for adenovirus [25] and other respiratory viruses (human metapneumovirus, human parainfluenza viruses 1 to 3, influenza B and respiratory syncytial virus [RSV]) using CDC rRT-PCR protocols (D. Erdman, pers. comm.). A rapid antigen test (BinaxNow™, Inverness Medical Professional Diagnostics, Princeton, NJ) to detect infection with *Streptococcus pneumoniae* was used in urine samples from all persons >10 years of age.

### Data management and analysis

Data were collected using preprogrammed, hand-held personal digital assistants. Only data from 2008 and 2009 were included in this report. SAS v. 9.1 (Cary, NC) was used for analysis. The case fatality proportion (CFP) was calculated as the number of deaths among the cases of influenza A that occurred during hospitalization or within six weeks of discharge divided by the total number of cases of influenza A among hospitalized pneumonia patients. For non-normally distributed continuous variables, the Mann-Whitney U-test was used when two influenza subtypes were compared, and the Kruskal-Wallis analysis of ranks was used when three influenza subtypes were compared. Pearson's chi-square statistic or Fisher's exact test were used to test for differences in categorical variables between patients infected with different influenza A virus subtypes and a *P* value of <0.05 was considered statistically significant. Variables with missing data for >10% of the patients were analyzed to ensure no difference in terms of age, sex or influenza A subtype between those with and without data.

## Results

Between January 1, 2008 and December 31, 2009, we identified 1744 eligible hospitalized pneumonia patients and enrolled 1666 (96%) in the surveillance system; respiratory samples were obtained and tested from 1602 (96%) of those enrolled. During the same period, we identified 899 eligible ILI patients, enrolled 801 (89%), and obtained and tested samples from 793 (99%) of those enrolled. There were no significant differences in either sex or age distributions between the eligible pneumonia or ILI patients who consented to enrollment and those who did not, or between those enrolled pneumonia or ILI patients who agreed to have a respiratory specimen taken and those who declined (data not shown).

**Figure 1:**
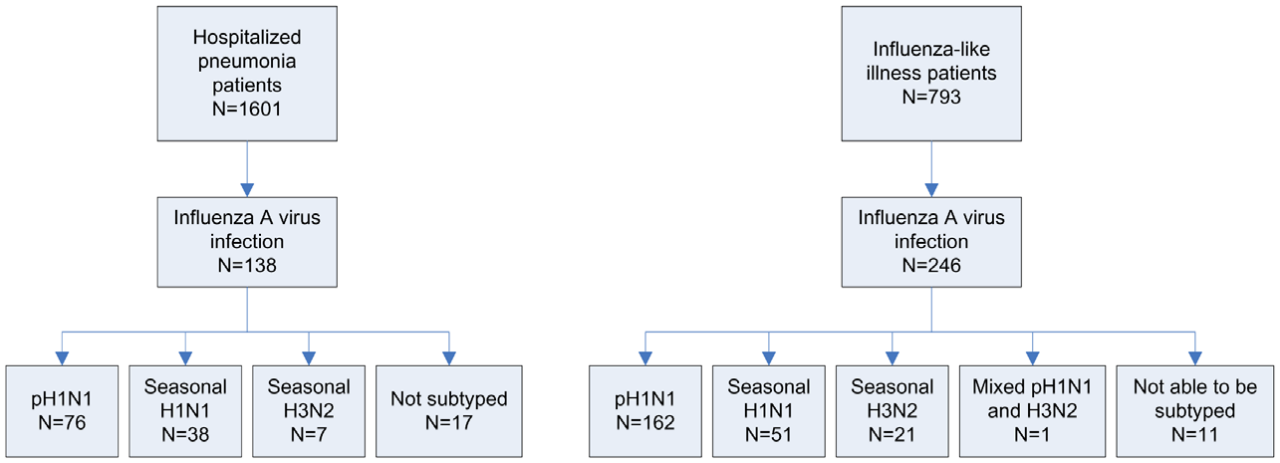
Study Profile, Guatemala, 2008-2009.

There were 138 (9%) hospitalized pneumonia and 246 (31%) ILI patients with laboratory-confirmed influenza A virus infection (Figure 1). The pH1N1 virus was the most common influenza A virus subtype identified among both the pneumonia (76/138, 55%) and ILI (162/246, 66%) patients. Mixed pH1N1 and H3N2 virus infection occurred in one (0.4%) case of ILI. Of the 17 (12%) samples positive for influenza A virus from pneumonia patients and 11 (4%) from ILI patients that we were unable to subtype, all but three occurred during the pandemic period. Among the influenza A viruses that could be subtyped, there was no significant difference in the subtype distribution between pneumonia and ILI patients (*P* = 0.17).

As a result of the small number of H3N2 in the hospitalized pneumonia patients, we have limited comparison of the characteristics of hospitalized pneumonia patients to those with seasonal H1N1 and pH1N1.

**Figure 2:**
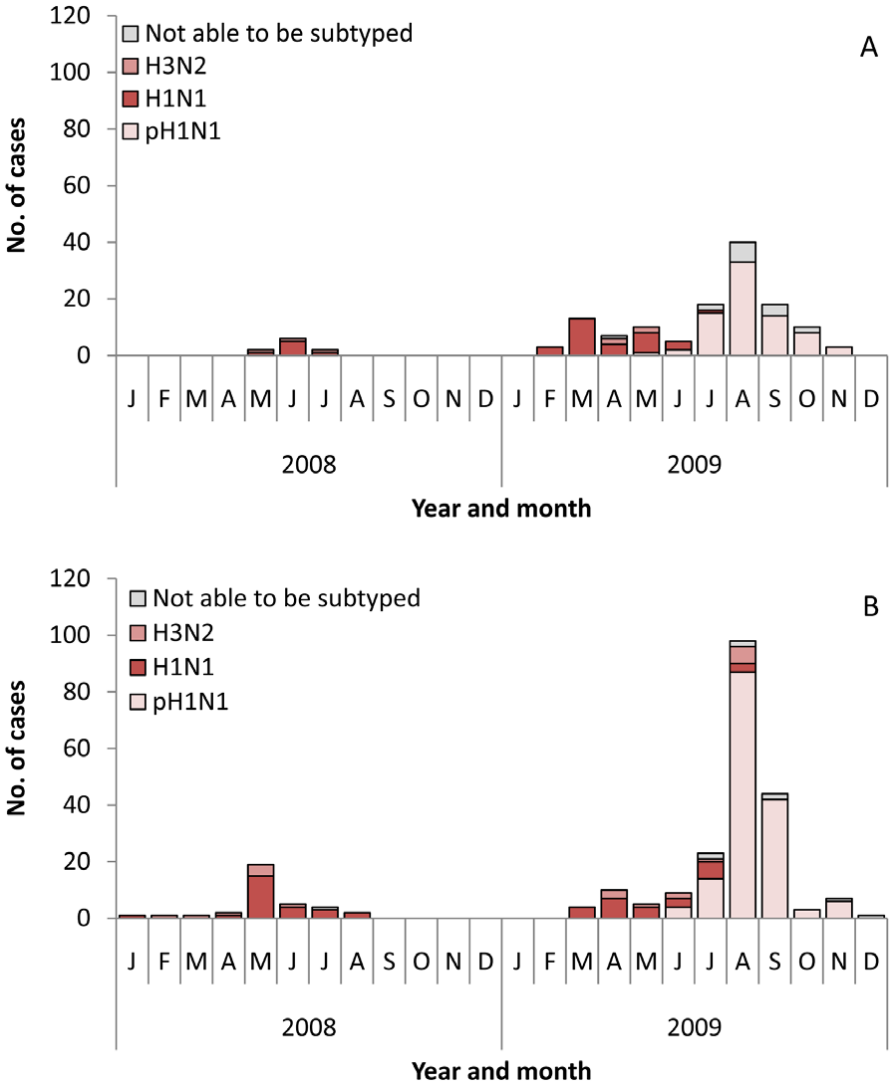
Number of Patients with Influenza A by Month and Subtype, Guatemala, 2008-2009. Panel A: Hospitalized pneumonia patients. Panel B: Influenza-like illness patients.

During 2008, surveillance for hospitalized pneumonia and ILI was underway only in Santa Rosa; all cases of seasonal influenza A during 2008 occurred between January and August, with the peak number of cases, both of hospitalized pneumonia (Figure 2a) and ILI (Figure 2b), presenting between May and July. Thirty-three (72%) of the cases of influenza A in 2008 were of the seasonal H1N1 subtype. In 2009, cases of influenza were reported beginning in February. There was an early peak of seasonal H1N1 and H3N2 from March to May; however, the second peak due to the pH1N1 virus, from June through September, was greater. In 2009, 238 (70%) of the cases of influenza A were of the pH1N1 subtype. There were 83 (57%) cases of seasonal influenza A that occurred prior to the pandemic.

**Figure 3:**
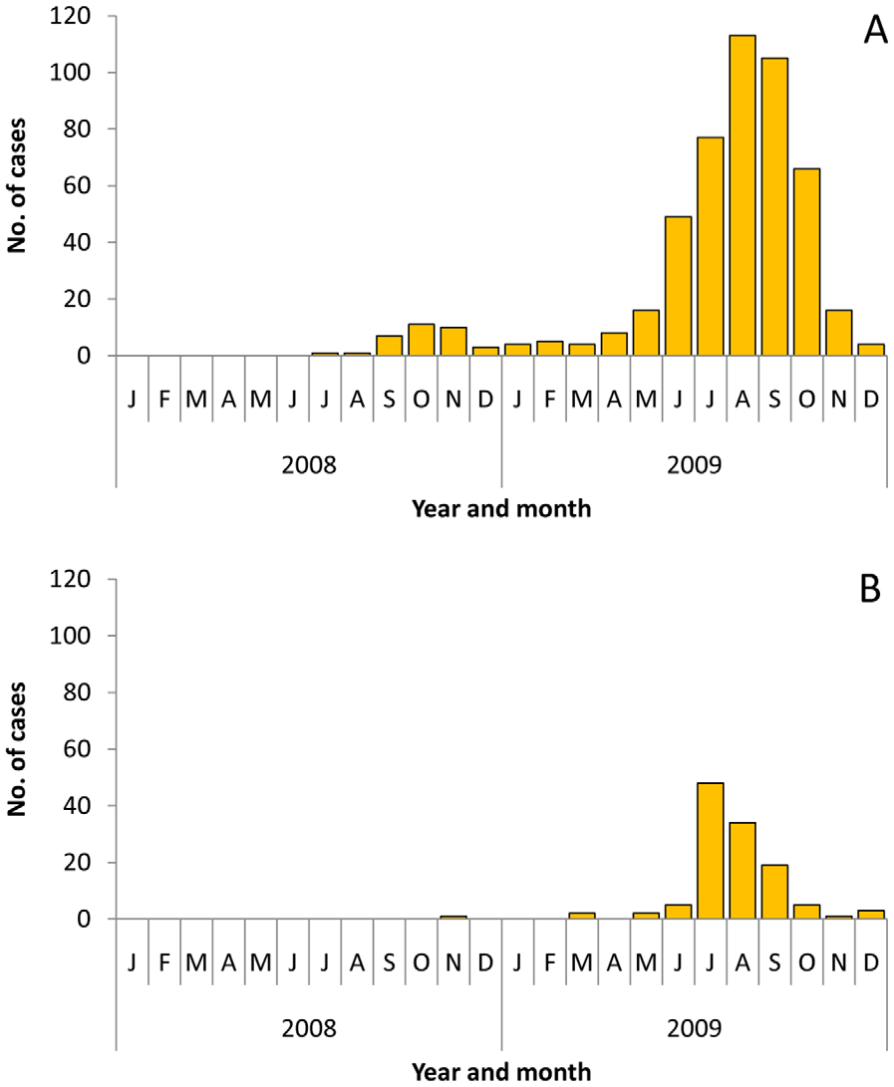
Number of Patients with Respiratory Syncytial Virus by Month, Guatemala, 2008-2009. Panel A: Hospitalized pneumonia patients. Panel B: Influenza-like illness patients.

Concomitant with the 2009 influenza pandemic was a significant increase in the number of RSV-associated respiratory infections, which peaked in July and August (Figure 3).

### Age distributions

**Figure 4:**
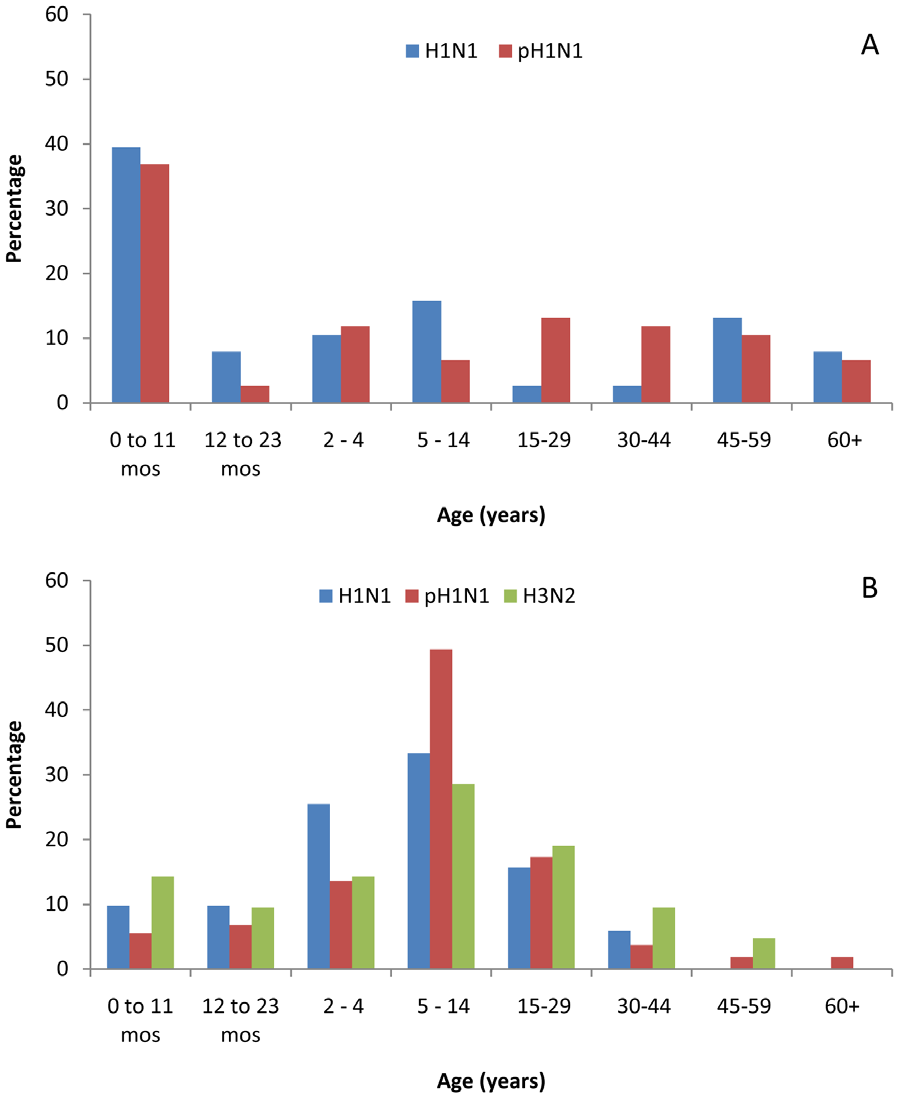
Age Distribution of Patients by Influenza A Subtype, Guatemala, 2008-2009. Panel A: Hospitalized pneumonia patients. Panel B: Influenza-like illness patients.

The median age of hospitalized pneumonia patients with influenza A was 3 years, and was lower for seasonal H1N1 (2 years) than for pH1N1 (4 years), but the difference was not statistically significant (*P = *0.48) (Table 2). The proportions of hospitalized pneumonia patients aged <1 year old with seasonal H1N1 (15/38; 39%) and pH1N1 (28/76; 37%) were similar (*P* = 0.42) (Figure 4a). Young adults 15 to 29 years old made up a larger proportion of hospitalized pneumonia patients with pH1N1 (10/76; 13%) than seasonal H1N1 (1/38; 3%) but the difference was not statistically significant (*P* = 0.1). The proportion of hospitalized pneumonia patients 60 years or older with seasonal H1N1 (3/38; 8%) and pH1N1 (5/76; 7%) were similar (*P* = 1.0). Overall, there was no significant difference in the age distribution of hospitalized pneumonia patients between seasonal H1N1 and pH1N1 (*P = *0.21).

**Table 2:** Characteristics of Pneumonia Patients by Influenza A Subtype– Guatemala, 2008-2009.

	All influenza A**No. (%)*	Influenza A subtypeNo. (%)*		
Characteristics	N = 138	Seasonal H1N1N = 38	pH1N1N = 76	P value
Age in years, median (IQR)	3.0 (0.7–38)	2.3 (0.7–25)	4.2 (0.7–37)	0.48
Sex, F	55 (40)	14 (37)	31 (41)	0.68
Previously sought care for this illness	71 (56)	20 (63)	45 (59)	0.75
Days from symptom onset to admission, median (IQR)	5 (3–8)	4 (3–8)	5 (4–8)	0.27
Took any medication before admission	88 (64)	26 (68)	53 (70)	0.89
Took antipyretics before admission	67 (50)	22 (58)	40 (55)	0.76
Took antivirals before admission	3 (2)	0 (0)	3 (4)	0.55
Clinical signs and symptoms at admission				
Cough	129 (95)	33 (92)	75 (99)	0.06
Difficulty breathing	115 (85)	31 (86)	66 (87)	0.91
Shortness of breath†	67 (82)	16 (80)	38 (83)	0.80
Sputum production	96 (71)	28 (78)	52 (68)	0.31
Sore throat†	49 (65)	11 (65)	32 (73)	0.54
Fever‡	97 (70)	32 (84)	47 (62)	0.01
Rhinorrhea	85 (62)	23 (62)	50 (66)	0.71
Wheezing	84 (62)	25 (66)	44 (60)	0.51
Headache†	51 (61)	14 (64)	29 (63)	0.70
Pleuritic chest pain†	42 (59)	10 (53)	26 (65)	0.36
Tachypnea	76 (56)	27 (71)	42 (57)	0.14
Pulse ox <90%	57 (51)	14 (58)	37 (53)	0.64
Myalgia†	37 (47)	9 (41)	24 (52)	0.68
Chills	58 (43)	15 (40)	34 (45)	0.26
Diarrhea	25 (18)	10 (26)	13 (17)	0.25
Chest radiograph consistent with pneumonia	47 (64)	14 (56)	27 (71)	0.22
Underlying medical conditions				
One or more conditions	26 (19)	8 (16)	14 (18)	0.93
Asthma	10 (8)	4 (9)	4 (5)	0.47
Lung disease	3 (2)	1 (2)	2 (3)	0.84
Diabetes	5 (4)	3 (7)	1 (1)	0.13
Chronic cardiovascular diseasê	7 (5)	2 (5)	5 (7)	0.37
Immunocompromised	1 (1)	0 (0)	0 (0)	—
Pregnancy§	2 (29)	0 (0)	2 (17)	0.53
Current smoker	7 (13)	2 (20)	5 (16)	0.98
Coinfection with other respiratory virus	31 (22)	7 (18)	17 (22)	0.63
Respiratory syncytial virus	16 (12)	0 (0)	11 (15)	0.01
Adenovirus	14 (10)	5 (14)	7 (9)	0.49
Coinfection with *Streptococcus pneumonia*e¥	11 (22)	2 (22)	6 (19)	1.0

Note: IQR  =  interquartile range.

*Percentages are a proportion of non-missing data.

**Includes 17 patients with influenza A that could not be subtyped, and seven patients with influenza A (H3N2).

† Only assessed in patients ≥2 years old.

‡ Measured temperature ≥38°C within first 24 hours of admission.

§ Only evaluated in females ≥14 and <45 years old.

^Only evaluated in those ≥15 years old.

¥Only evaluated in those ≥10 years old.

The age distribution of the ILI patients with influenza A differed from that of hospitalized pneumonia patients (Figure 4b) (*P*<0.001), with the median age of ILI patients (8 years) greater than that for hospitalized pneumonia patients (3 years). Among ILI patients, there was no statistical difference between the age distribution of pH1N1 and seasonal H1N1 (*P* = 0.22) or pH1N1 and H3N2 (*P = *0.25) (Table 3). The proportion of ILI patients with pH1N1 that was <1 year old (20/162, 12%) was not significantly different from the proportion of ILI patients with either seasonal H1N1 (10/51, 20%; *P* = 0.25) or H3N2 (5/21, 24%; *P* = 0.17). Similarly, the proportion of ILI patients with pH1N1 that were ≥60 years old (3/162, 2%) was not significantly different from seasonal H1N1 (0/51, 0%; *P* = 1.0) or H3N2 (0/21, 0%; *P = *1.0). The group most affected by ILI due to influenza A was school-age children from five to 14 years old with 108 (44%) cases of influenza A; there was no statistically significant difference in the proportion of pH1N1 cases aged 5 to 14 years old (80/162, 49%) in comparison to seasonal H1N1 (17/51, 33%; *P = *0.05) or H3N2 (6/21; 29%; *P* = 0.10).

**Table 3:** Characteristics of Patients with Influenza-Like Illness by Influenza A Subtype – Guatemala, 2008–2009.

	All influenza A*No. (%)**	Influenza A SubtypeNo. (%)**			
Characteristics	N = 246*	Seasonal H1N1N = 51	H3N2†N = 21	pH1N1†N = 162	P value
Age, median (IQR)	8 (4–15)	7 (3–14)	7 (3–22)	9 (5–15)	0.24
Sex, Female	116 (47)	26 (51)	12 (57)	72 (44)	0.45
Previously sought care for this illness	50 (23)	11 (32)	4 (31)	34 (21)	0.61
Days from symptom onset to presentation, median (IQR)	2 (1–3)	2 (1–3)	2 (1–2)	2 (1–3)	0.11
Clinical signs and symptoms at presentation					
Fever‡	246 (100)	51 (100)	21 (100)	162 (100)	1.0
Cough	233 (95)	49 (96)	18 (86)	155 (96)	0.13
Headache∼	193 (87)	44 (92)	14 (88)	125 (86)	0.82
Sore throat∼	191 (86)	45 (92)	15 (94)	121 (84)	0.22
Myalgia∼	169 (77)	39 (81)	13 (81)	109 (75)	0.65
Chills	181 (74)	39 (77)	15 (71)	117 (75)	0.94
Rhinorrhea	168 (69)	39 (78)	13 (62)	111 (69)	0.31
Sputum production	121 (50)	30 (60)	13 (62)	75 (47)	0.14
Pleuritic chest pain∼	97 (45)	30 (64)	10 (67)	53 (37)	0.001
Difficulty breathing	101 (41)	30 (59)	12 (57)	55 (34)	0.002
Shortness of breath∼	53 (24)	11 (23)	4 (25)	35 (25)	0.97
Diarrhea	21 (9)	7 (14)	1 (5)	11 (7)	0.24
Coinfection with respiratory virus	29 (12)	7 (14)	3 (14)	15 (9)	0.57
Respiratory syncytial virus	18 (7)	0 (0)	2 (10)	12 (7)	0.12
Adenovirus	7 (3)	5 (10)	0 (0)	2 (1)	0.005

Note: IQR  =  interquartile range.

*The total number of patients with influenza A includes one mixed pH1N1 and H3N2 infection, and 11 influenza A samples that could not be subtyped.

**Unless indicated otherwise, percentages are a proportion of non-missing data.

† Does not include one patient with a mixed pH1N1 and H3N2 infection.

‡ Measured temperature >38°C.

∼ Only assessed in patients ≥2 years old.

### Differences in clinical presentation of influenza A cases by age

Children <5 years old admitted with pneumonia and found to have influenza A were more likely to have sought care previously (42/67, 63%) than persons ≥5 years old (29/61, 48%), but the difference was not statistically significant (*P* = 0.09). There was no difference in the proportion of children <5 years old and persons ≥5 years old with respect to having taken any medication, including antipyretics and antivirals, within the 72 hours prior to admission (data not shown). Children <5 years old admitted with pneumonia were more likely to present with wheezing (55/72, 76%) than persons ≥5 years old (29/64, 45%; *P* = 0.0002), but were less likely to present with tachypnea (persons <5 years old: 28/71, 39%; persons ≥5 years old: 48/65, 74%; *P*<0.0001). Headaches, muscle aches and shivering were significantly more common among persons ≥5 years old admitted with pneumonia compared to children <5 years old (data not shown). There was no significant difference by age in the proportion of hospitalized pneumonia patients with cough, difficulty breathing, shortness of breath, sputum production, sore throat, fever, rhinorrea, pleuritic chest pain, oxygen saturation <90% or diarrhea. Children <5 years old were more likely to be coinfected with another respiratory virus (27/73, 37%) than were persons ≥5 years old (5/65, 8%; *P*<0.001). There were no significant differences in severe outcomes (admission to the ICU, mechanical ventilation or death) between children <5 years old and persons ≥5 years old (data not shown).

Among ILI patients with laboratory-confirmed influenza A, children <5 years old (12/56, 21%) were no more likely to have sought care elsewhere prior to presentation at the ambulatory clinic than persons ≥5 years old (33/139, 24%; *P = *0.73). Children <5 years old were more likely to present with cough (65/65, 100%) than persons ≥5 years old (139/148, 94%; p = 0.04). Diarrhea was also more commonly reported from children <5 years old (10/65, 15.4%) than persons ≥5 years old (8/148, 5%; p = 0.02). Patients ≥5 years old were significantly more likely to report muscle aches and shivering than patients <5 years old (data not shown). There was no difference in the proportion of children <5 years old and persons ≥5 years old in terms of viral coinfection (data not shown). Among ILI patients, there were no significant differences in the presence of cough, sore throat, rhinorrhea, sputum production, pleuritic chest pain, difficult breathing or shortness of breath by age (data not shown).

### Characteristics and clinical presentation of pneumonia patients by influenza A subtype

About 40% of the influenza A cases were females, and there was no significant difference in sex between pH1N1 and seasonal H1N1 (*P = *0.68; Table 2). Treatment-seeking behavior and treatment received prior to hospital admission for pneumonia was similar between patients with seasonal H1N1 and pH1N1. The interval between symptom onset and hospital admission was similar for both subtypes, between four and five days.

Clinical presentation of hospitalized pneumonia patients with influenza A was similar between patients infected with seasonal H1N1 and pH1N1; cough, difficulty breathing and shortness of breath were the most common symptoms reported by patients with both subtypes (Table 2). However, a measured temperature ≥38°C within the first 24 hours of admission was less frequent among hospitalized pneumonia patients with pH1N1 (62%) than among those with seasonal H1N1 (84%; *P = *0.01). We did not evaluate the frequency of vomiting or nausea among these patients, but there was no difference in the proportion of patients reporting diarrhea by subtype (*P = *0.25).

As reported previously [22], in this population, the proportion of hospitalized pneumonia patients with influenza A reporting a chronic or underlying medical problem was very low, less than 20%, and there was no significant difference between patients with seasonal H1N1 and pH1N1 (*P = *0.93). Asthma was the most common condition reported. There were no women aged 14 to 44 years old hospitalized with seasonal H1N1; more than a quarter (29%) of the female patients with pH1N1 in this age range was pregnant, but this amounted to only two women.

Almost a quarter (22%) of hospitalized pneumonia patients with influenza A were admitted to the ICU (Table 4). This proportion was higher for patients infected with pH1N1 (28%) than for seasonal H1N1 (18%) but the difference was not statistically significant (*P = *0.28). We examined the proportion of hospitalized pneumonia patients with influenza A admitted to the ICU before the pandemic began in May 2009 (7/34, 21%) and after (23/104, 22%), but there was no statistical difference (*P = *0.85). The proportion of patients with respiratory distress who required mechanical ventilation was more than three times higher among the hospitalized pneumonia patients with pH1N1 (11%) compared with those with seasonal H1N1 (3%), but the difference was not statistically significant (*P = *0.14).

**Table 4:** Outcomes of Pneumonia Patients by Influenza A Subtype– Guatemala, 2008–2009.

	All influenza A*No. (%)**	Influenza A subtypeNo. (%)**		
Outcome	N = 138	Seasonal H1N1N = 38	pH1N1N = 76	P value
Admitted to the ICU	30 (22)	7 (18)	21 (28)	0.28
Mechanical ventilation	9 (7)	1 (3)	8 (11)	0.14
Death†	13 (9)	2 (5)	11 (15)	0.14
Length of hospital stay, days, median (IQR)	5 (3–7)	5 (4–7)	5 (2–9)	0.41
Symptom onset to death, days, median (IQR)	9 (7–16)	15.5 (15–16)	9 (6–16)	0.23
Age in years at death, median (IQR)	26 (0.9–54)	56 (55–57)	15 (0.9–50)	0.07

Note: ICU  =  intensive care unit; IQR  =  interquartile range.

*Includes 17 patients with influenza A that could not be subtyped, and seven patients with influenza A (H3N2).

**Percentages are a proportion of non-missing data.

† Includes two hospitalized pneumonia patients who died one day after being discharged from the hospital.

The CFP was three times higher for hospitalized pneumonia patients with pH1N1 (15%) compared to seasonal H1N1 (5%), but this difference was not statistically significant (*P = *0.21) (Table 4). The two deaths associated with seasonal H1N1 both occurred in patients between 50 and 59 years of age during their hospitalization. Out of the 11 deaths among patients with pH1N1, nine occurred in the hospital and two within one day of discharge. Four of the patients with pH1N1 who died were <1 year old, two were between one and 15 years old, two were between 20 and 49 years old, and three were between 50 and 59 years old. There were no deaths of patients with pH1N1 ≥60 years old.

Coinfection with another respiratory virus was similar between hospitalized pneumonia patients with seasonal H1N1 and pH1N1 (*P = *0.52) (Table 2). However, there were different respiratory viruses associated with coinfection by influenza A subtype: adenovirus was the most common viral coinfection among the hospitalized pneumonia patients with seasonal H1N1, with 13% of the patients coinfected. In contrast, RSV was the most common viral coinfection among hospitalized pneumonia patients with pH1N1, coinfecting 14% of these patients. The significant difference in the proportion of patients with pH1N1 compared with seasonal H1N1 coinfected with RSV was due to a much greater incidence of RSV in 2009 than in 2008, with the peak number of cases coinciding with the 2009 influenza pandemic (Figure 3).

Among the 39 hospitalized pneumonia patients <5 years old with pH1N1, there was no difference in the proportion admitted to the ICU between those patients coinfected with RSV (3/10, 30%) than those not coinfected with RSV (9/29, 31%; *P = *1.0). A greater proportion (2/10, 20%) of the patients coinfected with RSV than the patients not coinfected with RSV (2/29, 7%) required mechanical ventilation, but the difference was not statistically significant (*P = *0.27). Similarly, the CFP among patients coinfected with RSV (3/10, 30%) was higher than that among those not RSV coinfected (2/29, 7%), but the difference was not statistically significant (*P = *0.1). Of the four deaths associated with pH1N1 among this age group, three (75%) patients were coinfected with RSV.

There was no difference in the proportion of children and adults ≥10 years old who were coinfected with *S. pneumoniae* by subtype (Table 2).

### Clinical presentation and characteristics of patients with ILI

Less than a quarter (23%) of the ILI patients with influenza A sought care outside the home before presenting at the health center or health post, and this percentage did not differ significantly by influenza A subtype (*P = *0.61) (Table 3). The median number of days from onset of symptoms to presentation at an ambulatory clinic for patients with influenza A was two days and was similar across all influenza subtypes (*P* = 0.11).

Clinical signs and symptoms of patients with ILI were similar across influenza A subtypes (Table 3), apart from difficulty breathing and pleuritic chest pain, which were significantly less likely to be reported by ILI patients with pH1N1 than those with seasonal H1N1 or influenza A (H3N2). We found a higher proportion of patients with diarrhea among those with seasonal H1N1 (14%) than either pH1N1 (7%) or H3N2 (5%), although this difference was not statistically significant (*P* = 0.24).

The proportion of ILI patients with influenza A who were coinfected with another respiratory virus was higher among ILI patients with seasonal H1N1 (14%) and H3N2 (14%) than pH1N1 (9%), but the difference was not statistically significant (*P = *0.57). Whereas the proportion of ILI patients with pH1N1 who were coinfected with RSV (7%) was higher than the proportion of seasonal H1N1 (0%), the proportion of ILI patients with H3N2 coinfected with RSV was similar (10%), and there was no overall difference by influenza A subtype (*P* = 0.12).

## Discussion

Although the 2009 influenza pandemic caused significant concern in Guatemala and helped to increase recognition of influenza as an important public health issue, the data we have presented here suggest that in Guatemala, the clinical presentation of pH1N1 was similar to that of the seasonal influenza viruses that were circulating before and during the pandemic.

Despite reports of significant differences in the age distributions of seasonal influenza A and pH1N1 in both temperate [8], [26]–[27] and tropical climates [16], [18], we found similar age distributions for both ILI and pneumonia associated with seasonal influenza A and pH1N1. Both seasonal H1N1 and pH1N1 caused pneumonia primarily in children <1 year old; infants account for approximately 3% of the Guatemalan population, but they made up 37% and 39% of the hospitalized pneumonia patients with pH1N1 and seasonal H1N1, respectively. Among ILI patients, school-age children 5 to 14 years old accounted for a third to half of influenza A cases, and they were the predominant age group affected by all three influenza A virus subtypes.

Although not statistically significant, all indicators of severity (i.e., admission to an ICU, mechanical ventilation and CFP) were higher among hospitalized pneumonia patients with pH1N1 as compared with seasonal H1N1. Because our study combined pre-pandemic and pandemic periods, we analyzed whether changes in practices or procedures could have resulted in findings of greater severity for pH1N1 than seasonal influenza. The proportion of hospitalized pneumonia patients admitted to the ICU was similar before and after the pandemic began, and although use of antivirals was rare, treatment with antivirals only occurred during the pandemic period. There was no difference between hospitalized pneumonia or ILI patients in time to presentation at a health facility by influenza A subtype.

There have been three other concurrent comparisons of seasonal influenza and pH1N1 in hospitalized patients that reported on severe outcomes; none found any significant differences in the ICU admission rates or CFP by influenza subtype, but all occurred in well-resourced settings where antivirals would have been available for treatment [13]–[15]. We report a higher CFP for pH1N1 than has been reported elsewhere, but this is likely due, in part, to a limited supply of antivirals available for treatment, and does not explain the higher CFP for pH1N1 than seasonal H1N1.

One possible explanation for the higher CFP from pH1N1 in Guatemala is the increase in RSV transmission during the pandemic period. Viral coinfection, especially with RSV, has been hypothesized to reduce the T helper cell 1 response, thereby increasing disease severity [28]. Among the children <5 years old with pH1N1 who died, 75% were coinfected with RSV. It is not clear whether RSV acted synergistically with pH1N1 to cause more severe disease in these patients, or whether RSV itself might have been the cause of their death. Further investigation in this population of the effect of RSV and influenza coinfection is warranted.

Clinical symptoms of hospitalized pneumonia patients were similar between seasonal H1N1 and pH1N1, except for measured temperature ≥38°C in the first 24 hours after hospital admission, which was significantly less frequent among patients with pH1N1. We looked for differences in treatment seeking behaviors and treatments taken before admission that could explain this finding, but the use of any medication, and antipyretics in particular, did not differ between hospitalized pneumonia patients with seasonal H1N1 and pH1N1. A difference in fever has not been noted in any other concurrent comparison of patients with seasonal influenza A and pH1N1 [13]–[15].

Although there have been many reports of a higher proportion of pH1N1 ILI patients with gastrointestinal symptoms [8]–[10], [16], we found no difference in the proportions of hospitalized pneumonia or ILI patients with diarrhea by subtype. It is possible that our use of a stringent case definition for diarrhea may have missed an association with more mild gastrointestinal symptoms such as nausea.

A recent comparison of seasonal influenza and pH1N1 cases in Philadelphia found more lower respiratory tract symptoms (i.e., cough and pleuritic chest pain) among pH1N1 than seasonal influenza cases [14]; we did not find any significant differences in the prevalence of these symptoms among hospitalized pneumonia patients, but among ILI patients, both difficulty breathing and pleuritic chest pain were significantly more common among seasonal influenza patients, rather than those with pH1N1. It is possible that during the pandemic, patients with lower respiratory tract symptoms were more likely to proceed directly to the hospital for treatment.

This study has several important strengths. Case definitions, laboratory diagnostics and procedures for data collection did not change during the time period covered in this report; this eliminates the possibility that findings were related to changes in surveillance methodology as a result of the pandemic, which can be a problem when using historical controls. A broad case definition permitted inclusion of influenza cases that might otherwise go undetected; for example, requirement of fever in the case definition for severe acute respiratory disease could miss a significant proportion of serious illness associated with both seasonal H1N1 and pH1N1.

The main limitation of this study is the relatively small number of cases of influenza that could be analyzed, which limits the power to detect differences in characteristics and clinical presentation. Because the sample size was small, it is possible that we were not able to identify important differences between seasonal influenza A viruses and pH1N1 influenza that might appear in a larger data set, especially related to signs of severity which were consistently elevated with pH1N1 but were not statistically significant. This limitation has been noted for least one other similar study [29], and should be taken into consideration when evaluating results from our study. Because surveillance for pneumonia in Quetzaltenango was initiated only four months before the pandemic began, and surveillance for ILI was initiated two months after the first case of pandemic influenza, the majority of the seasonal influenza cases come from Santa Rosa and this may have introduced some unmeasured biases in the comparison between seasonal influenza and pH1N1. Although we did not find a difference in the number of days between symptom onset and care seeking at our surveillance clinics and hospitals between seasonal influenza A viruses and pH1N1, we are unable to determine from this dataset whether there was an increase in the probability of healthcare seeking as a result of the pandemic. We used a standard definition for ILI that includes a measured fever and this is likely to have caused us to miss cases of influenza that presented without fever.

In conclusion, the epidemiology of pH1N1 in Guatemala was not significantly different from that associated with the seasonal influenza subtypes circulating locally before and during the pandemic in terms of the age groups most affected and clinical signs and symptoms. In Guatemala, influenza is largely a disease of children, with the most severe disease in infants, and targeted use of influenza vaccine in children may be warranted. The 2009 influenza pandemic raised awareness of the burden of disease caused by influenza in the tropics; increased attention should be extended to monitoring and addressing the morbidity and mortality associated with seasonal influenza.
